# Editorial: Motivations for physical activity—Volume III

**DOI:** 10.3389/fpsyg.2025.1666422

**Published:** 2025-08-13

**Authors:** Pedro Morouço, Aleksandra Maria Rogowska, Iuliia Pavlova

**Affiliations:** ^1^National Program for the Promotion of Physical Activity, Directorate-General of Health, Lisbon, Portugal; ^2^Faculty of Social Sciences, Institute of Psychology, University of Opole, Opole, Poland; ^3^Lviv State University of Physical Culture, Lviv, Ukraine

**Keywords:** physical activity, sport, motivation, psychology, technology

## Introduction

The practice of regular physical activity is widely recognized as one of the main pillars of health throughout the life cycle. Despite the robust evidence supporting its benefits (e.g., in the prevention of chronic diseases, in the improvement of mental health, in the promotion of wellbeing and longevity) a significant part of the population still does not comply with the minimum recommendations for physical activity. This paradox between knowledge and action has motivated increasing attention from the international scientific community, seeking to understand the psychological and behavioral mechanisms that influence, facilitate or inhibit the regular practice of physical activity and sports participation.

It was in this context that this Research Topic was born, jointly hosted by the journals Frontiers in Psychology and Frontiers in Sport and Active Living, with the aim of aggregating scientific contributions that explore the psychological and behavioral dimensions of the practice of physical activity and involvement in sport, at different ages, cultures, contexts and modalities. The set of 20 articles gathered in this Research Topic constitutes a valuable repository of empirical data, theoretical reflections, and proposals for concrete intervention applicability. This editorial thus aims to present a critical synthesis of the main emerging themes, articulate individual contributions in an integrated narrative and highlight the implications for research, practice and public policy formulation. The methodological and geographical diversity of the studies presented allows us to observe more clearly the complexity of this phenomenon and the way in which different variables interact, reinforce, or limit the active involvement of people throughout the various stages of life. Contributions range from psychometric analyses to qualitative investigations, from clinical trials to systematic reviews, offering a comprehensive and interdisciplinary panorama.

## Motivation, identity and self-regulation: structuring nuclei

Motivation continues to be one of the most studied axes in the field of physical activity, with approaches ranging from classical theories (e.g., self-determination, planned behavior) to more recent perspectives, focused on hope, grit or athletic identity.

Kovács analyses motivational profiles of more than a thousand young Hungarian athletes, highlighting the complexity of the interactions between sports orientation, persistence and personality traits. The study by Tokarska and Rogowska, used a person-centered approach to identify distinct motivational profiles in cyclists and runners. The authors revealed that different combinations of intrinsic and extrinsic motivation, as well as levels of self-efficacy, configure typologies that directly impact the behavior of athletes. This differentiated vision allows for the design of intervention strategies that are more adjusted to the reality of each practitioner. The contribution of Ronca et al. broadens this reflection suggesting that personality traits (such as extraversion or emotional stability) can influence not only the preference for certain intensities of physical exercise, but also the magnitude of stress reduction obtained after a training program. This work highlights the importance of individualizing activity proposals, considering psychological predispositions. In the sphere of self-regulation, Blythe et al. demonstrate that hope agency predicts the ability to achieve exercise goals in college students. Participants with higher levels of hope showed greater consistency and success in achieving the set physical goals, pointing to the relevance of positive, future-oriented motivational states. Finally, Zhou et al. present a meta-analysis that reveals a moderate and bidirectional association between exercise motivation and cardiorespiratory fitness in students, consolidating the evidence of the importance of motivation for physical development and vice versa.

## Self-efficacy, confidence, and psychological wellbeing

The role of self-efficacy as a behavioral determinant is evidenced in several studies in this collection. The meta-analysis by Xie et al. demonstrates a consistent association between self-efficacy and physical activity in older adults, with a mutually reinforcing relationship. This virtuous cycle is especially relevant for the design of interventions aimed at active aging. The relevance of this construct also extends to other age groups, as evidenced by Kessler et al., who studied middle-aged and older adults with musculoskeletal pain. The work revealed that hope (understood as an emotional component related to self-efficacy) significantly predicts the levels of physical activity reported, even in the face of physical limitations. This finding reinforces the idea that positive psychological aspects can mitigate physical barriers and motivate active behavior. On the other hand, Na et al. contributed to the development and preliminary validation of a scale of parenting practices promoting physical activity for Chinese children between 3 and 6 years old. The application of this instrument revealed the significant influence of parenting styles on children's self-efficacy beliefs regarding movement and structured play practice, highlighting the importance of facilitating contexts from early childhood. Still in the field of the relationship between exercise and mental health, Zhang et al. demonstrated that regular physical activity in Chinese university students significantly improves emotional management, and this effect is mediated by two factors: life satisfaction and perceived health. These results point to psychological mediation mechanisms with potential for interventions in academic environments. Still in the field of the relationship between exercise and mental health, the study by Ahsan et al. analyzed the effects of physical activity on mental toughness and quality of life in a gender-differentiated sample of young adults. The authors identified differences between men and women in physical activity levels, as well as in several domains of mental strength and quality of life. These results reinforce the importance of considering gender as a moderating variable, and point to the role of physical activity as a transversal promoter of psychological skills and global wellbeing.

## Social factors, interpersonal relationships, and supportive climate

The influence of the social environment and interpersonal interactions is another theme that is strongly transversal to the articles included in this Research Topic. In fact, human behavior in the field of physical activity is profoundly influenced by the social environment. The quality of interpersonal relationships, leadership styles and the motivational climate play a decisive role in the way people start, maintain or abandon active practices. In performance sports, Liu et al. show that coaches' leadership behavior has direct and indirect positive effects (via coach-athlete relationship and psychological fatigue) on athletes' performance. This study reinforces the need for training in relational skills for training professionals. On the practitioners' side, Wineinger et al. explored effects of ego-centered motivational climates on master swimmers in the United States. The results indicate that excessively competitive environments, which promote social comparisons to the detriment of personal development, tend to compromise the perception of competence and long-term commitment. These data are particularly relevant in the context of senior sport, where wellbeing and active longevity should be priorities. Kim et al. show, in a study applied to the practice of Pilates, that subjective norms and perceived social value have greater weight than functional or emotional value in the intention to engage in practice. This evidence emphasizes the importance of perceived support and social influence in decision-making.

## Culture, values, and patterns of sports consumption

The practice of physical activity is not dissociated from cultural contexts and dominant value systems. They are also anchored in cultural values, social habits, and symbolic forms of belonging, expression, and consumption. Chen et al. use structural equation modeling to analyse the relationship between attitudes toward exercise and sports consumption patterns in Chinese urban residents. The results reveal that positive attitudes directly influence the practice, which, in turn, is associated with greater sports consumption; suggesting a circular model of behavioral and economic reinforcement. The article by Wambsganz et al. also contributes to this field, identifying intrapersonal factors (e.g., self-efficacy, intrinsic motivation, personality traits) that distinguish regular practitioners from inactive practitioners, in a sample of the German population. The study offers valuable clues for designing more effective behavioral campaigns.

## Technology, innovation, and emotional engagement

The incorporation of technology in the contexts of sports practice and physical activity has been profoundly transforming the way people engage with exercise. In addition to monitoring and personalization, the elements of immersion and emotional involvement have taken on an increasingly relevant role. For instance, the potential of technology as an enabler of active lifestyles is explored. In a pilot study Touloudi et al. compared a traditional exercise program with a virtual reality-based program in adults with obesity. The virtual reality intervention demonstrated greater effectiveness in improving body composition, activity levels, and exercise pleasure; key factors for sustained adherence. Emotionally and sensorily, Suwabe and Kawase reveal that music with high groove has positive effects on running speed and mood, especially among university women. These results show that immersive and pleasurable experiences can be catalysts for practice. The effectiveness of persuasive communication is addressed by Sun et al., who demonstrate that messages aligned with the recipient's regulatory style (promotion vs. prevention) are more effective in influencing the intention to exercise. This “regulatory fit” approach offers a promising avenue for more targeted and impactful campaigns.

Together, these studies underline that technological innovation and attention to emotional engagement are essential components for a contemporary approach to the promotion of physical activity. Technology should not be seen only as a measurement tool, but as a catalyst for positive, motivating and personalized experiences.

## Life transitions and behavioral changes

The practice of physical activity does not occur in a linear way throughout life. Transition phases, such as early adulthood, parenting, aging, or professional and social changes, are critical moments with a strong impact on movement-related behaviors. The transition to parenthood represents one of the most significant changes in adult life and has a direct impact on health behaviors. Bekhuis and van Abswoude analyse longitudinal data and conclude that after the birth of their first child, women tend to temporarily reduce their sports participation, while men maintain stable patterns. These data suggest that policies to promote physical activity should be sensitive to gender and family dynamics. At the opposite end of the life cycle, the study by González-Calvo et al. explored, through a qualitative approach with men between 65 and 76 years old, how physical practice contributes to the reconstruction of identity in aging. The authors identified that exercise promotes emotional wellbeing, challenges traditional norms of masculinity, and fosters meaningful social connections. Regular practice, especially in groups, emerges as a space of resistance to deficient narratives about aging, also evidencing the structural and socioeconomic barriers that limit access to physical activity for this age group. The research reinforces the importance of inclusive public policies and a positive and critical approach to active aging.

## Integration, diversity, and future implications

The set of articles gathered here represents a diverse and methodologically rich sample of research that focuses on the interface between psychology, human behavior and physical activity. By addressing different age groups, geographies, modalities and methodologies, this collection contributes to a more holistic and applied understanding of the phenomenon. The practical implications, which are numerous, can be summarized as: (i) interventions must be multicomponent, integrating individual (e.g., motivation, self-efficacy), contextual (social and physical environment) and technological (digital supports, sensory stimuli) factors; (ii) pleasure and emotional involvement should be considered not as a consequence, but as a condition that promotes adherence; (iii) the design of public policies must take an ecological perspective, articulating health, urbanism, education and culture; (iv) research should evolve toward longitudinally sustained approaches, with continuous measurement of effects and adaptation to social transformations (e.g., digitalisation, demographic aging, inequalities of access).

## Conclusion

This collection of twenty articles contributes significantly to the advancement of knowledge about the psychological and behavioral determinants of physical activity and sport. The studies presented here challenge traditional models, introduce innovative variables, validate approaches in different cultures and offer concrete clues for intervention. As editors, we express our sincere thanks to all the authors, reviewers, and contributors who made this publication possible. We believe that this Research Topic will not only be a reference for the scientific community, but also a catalyst for new ideas, programs and policies that are more effective in promoting active and healthy lifestyles.

